# Expansive-Type Breast Cancer Needs Careful Tissue Sampling to Avoid Needle Tract Recurrence: A Case Report

**DOI:** 10.7759/cureus.99482

**Published:** 2025-12-17

**Authors:** Karen Goto, Shoji Oura

**Affiliations:** 1 Department of Surgery, Kishiwada Tokushukai Hospital, Kishiwada, JPN

**Keywords:** breast cancer, core needle biopsy, expansive type, needle tract recurrence, viable cancer cell cluster

## Abstract

Needle tract recurrence in breast cancer is a rare form of recurrence, and there is limited data in the literature regarding its incidence. A 52-year-old nulliparous woman was referred to our hospital for the treatment of core needle biopsy-proven breast cancer. Mammography only showed a relatively well-defined mass. Ultrasound also showed a right breast mass, which displayed an expansive growth pattern. Magnetic resonance imaging of the main tumor showed low signals on T1-weighted images, high signals on T2-weighted images, and a washout pattern on dynamic studies. Consequently, the patient underwent a curative operation five weeks after the core needle biopsy. Postoperative pathological study showed atypical cells growing in a solid cribriform fashion, forming an expansive-type breast cancer measuring 17 mm, and isolated viable cancer cell clusters in the core needle biopsy-induced hematoma. Immunostaining showed estrogen and progesterone receptor positivity, human epidermal growth factor receptor type 2 negativity, and a Ki-67 labeling index of 20%. The patient received adjuvant radiotherapy to the conserved right breast, began taking an adjuvant aromatase inhibitor, and is scheduled to be followed up on an outpatient basis. Diagnostic physicians should be aware of the potential risk of needle tract recurrence associated with expansive-type breast cancers, especially in the case of core needle biopsies.

## Introduction

Breast cancer is the most common malignant disorder in women in many developed countries and generally shows favorable clinical outcomes compared with other malignant diseases [1]. Mammography and ultrasound are the mainstays in breast cancer diagnosis [2]. Diagnostic physicians further need the pathological diagnosis of breast cancer before performing surgery to avoid over-treatment.

Aspiration biopsy cytology has long been used to preoperatively diagnose breast cancer due to its minimally invasive characteristics [3]. Breast surgeons had exclusively performed mastectomies for nearly a century on breast cancer patients once the diagnosis of breast cancer was made. Previously, aspiration biopsy cytology played a very important role in breast cancer diagnosis due to the simplicity and low cost of the procedure. However, the advent of neoadjuvant chemotherapy has markedly reduced the clinical usefulness of aspiration biopsy cytology as a preoperative pathological examination [4].

Core needle biopsy, including vacuum-assisted biopsies, has enabled breast oncologists not only to make the definitive diagnosis of pathological malignancy, but also to develop therapeutic strategies by judging the breast cancer subtypes and aggressiveness using immunostaining. When adjuvant chemotherapy is considered imperative, oncologists now often treat even small breast cancers with negative lymph node metastasis, not with primary surgery but with neoadjuvant chemotherapy to aim for pathological complete response [5].

To our knowledge, needle tract recurrence has not been reported in breast cancer patients who preoperatively underwent aspiration biopsy cytology, but has sometimes been reported in those diagnosed by core needle biopsy [6]. This fact strongly suggests that more tissue sampling at the time of pathological examination is an important risk factor for needle tract recurrence. It further suggests that needle tract recurrence may occur in expansive-type, i.e., solid-type, breast cancer, possibly due to the harvesting of a greater number of cancer cells. Therefore, on performing core needle biopsy, it is important for physicians to consider the placement of biopsy puncture sites on the skin and whether the puncture site skin should be resected.

We herein report a case of expansive-type breast cancer in which pathological examination showed isolated viable cancer cell clusters moved from the original tumor by core needle biopsy, suggesting possible needle tract recurrence.

## Case presentation

**Figure 1:**
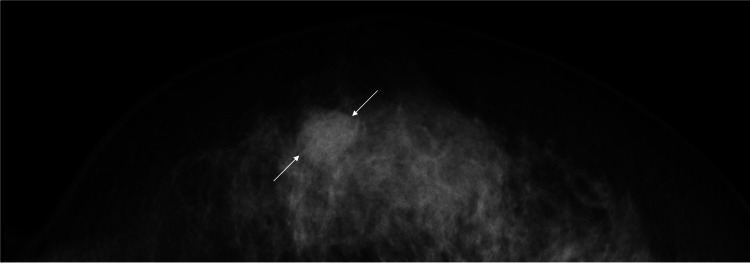
Mammography findings Mammography only showed a well-circumscribed mass (arrows) in the inner and lower part of her right breast.

A 52-year-old nulliparous woman who had undergone a hysterectomy and bilateral oophorectomy five years ago noticed a mass in her right breast and visited a breast clinic. The patient was thereafter referred to our hospital for breast cancer surgery due to the core needle biopsy-proven invasive ductal carcinoma. Mammography only showed a well-circumscribed mass within the dense breast (Figure 1).

**Figure 2:**
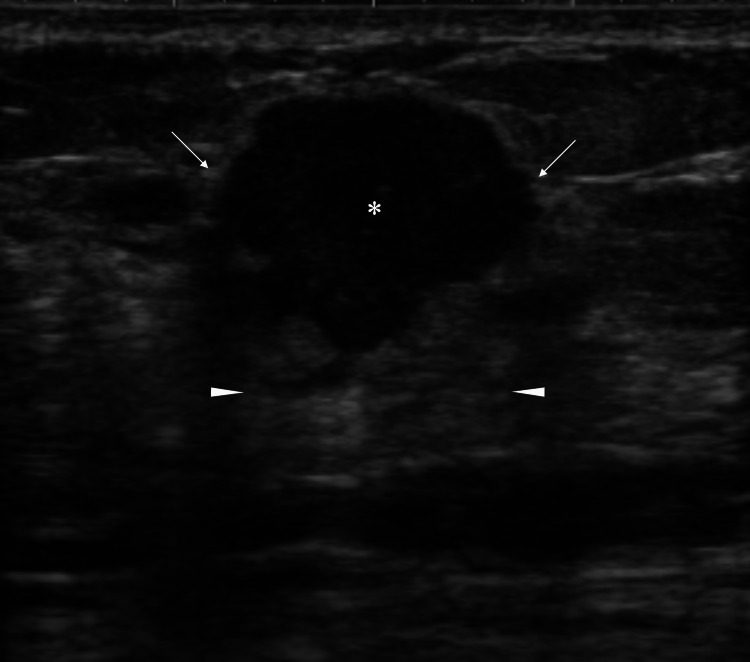
Ultrasound findings Ultrasound showed a polygonal tumor (asterisk) with internal low echoes, disruption of anterior borders of the mammary gland (arrows), enhanced posterior echoes (arrowheads),  and a tumor protrusion toward the overlying skin.

Ultrasound also showed a right breast mass which had distinct margins, a solid growth pattern, internal low echoes, enhanced posterior echoes, and protrusion close to the overlying skin (Figure 2).

**Figure 3:**
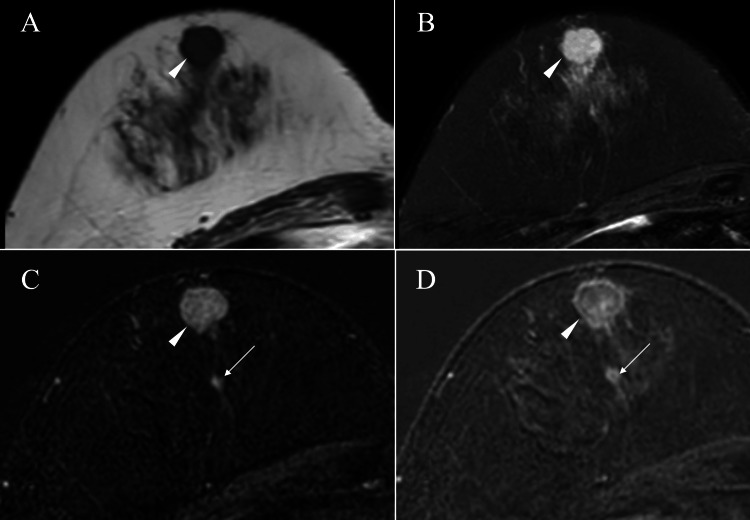
Magnetic resonance imaging (MRI) findings MRI of the main tumor showed low signals on T1-weighted images (A, arrowhead), high signals on fat-suppressed T2-weighted images (B, arrowhead), and a washout pattern on dynamic studies (C and D, arrowhead). MRI further showed a daughter nodule (C and D, arrow) in the peripheral areas of the main tumor.

Besides daughter nodules and ductal spread toward the nipple, magnetic resonance imaging (MRI) of the mass showed low signals on T1-weighted images, high signals on T2-weighted images, and a washout pattern on dynamic studies (Figure 3).

**Figure 4:**
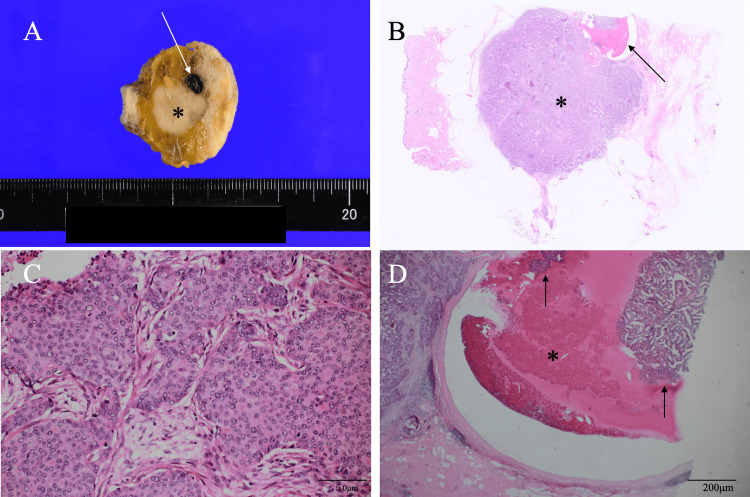
Pathological Findings A. Bisected cut surface showed a presumed needle biopsy-induced hemorrhage (arrow) adjacent to the tumor (asterisk). B. Low magnification view also showed an expansive type mass (asterisk) and core needle biopsy-induced hemorrhage (arrow). C. Magnified view clearly showed atypical cells growing in a solid manner. D. Magnified view showed at least two cancer cell clusters (arrows), disconnected to the main tumor, in the hemorrhage (asterisk).

Pathological study of the core needle biopsy specimen showed atypical cells growing in cribriform and solid fashion with comedo necrosis, leading to the diagnosis of invasive ductal carcinoma. The patient, therefore, underwent a partial mastectomy and sentinel node biopsy five weeks after the core needle biopsy. Despite the presence of micro-metastasis in the sentinel node on frozen section, the patient did not undergo axillary lymph node dissection according to the preoperative informed consent for the omission of axillary dissection. Postoperative pathological study showed atypical cells growing in solid and cribriform fashions, forming an expansive type breast cancer measuring 17 mm, isolated and viable cancer cell clusters in the needle biopsy-induced hematoma, and no cancer cell foci in the sentinel node (Figure 4). Immunostaining showed estrogen and progesterone receptor positivity, human epidermal growth factor receptor type 2 negativity, and a Ki-67 labeling index of 20%. Due to the patient's preference, the patient received adjuvant radiotherapy to the conserved right breast, began taking an aromatase inhibitor without receiving chemotherapy, and is scheduled to be followed up on an outpatient basis.

## Discussion

Adjuvant radiotherapy to the conserved breast in breast cancer patients can eradicate subclinical micro-cancer foci in the breast and therefore should also contribute to preventing the needle tract recurrence [7]. However, some breast cancer patients treated with breast-conserving surgery and adjuvant radiotherapy can develop needle tract recurrence, occasionally followed by distant metastasis, leading to dismal clinical outcomes [8]. Breast surgeons, therefore, should pay close attention to the needle tract recurrence on core needle biopsy, especially for breast cancers not planned for adjuvant radiotherapy.

To our knowledge, no prior studies have evaluated the pathological characteristics of the primary breast cancer in breast cancer cases developing needle tract recurrence to date. This patient had undergone breast cancer surgery five weeks after the core needle biopsy and pathologically had isolated viable cancer cell clusters, which were very similar to expansive-type main breast cancer and seemed to have migrated during the core needle biopsy procedures. It is naturally reasonable for breast oncologists to judge that five weeks are sufficient for breast cancer cells to develop some changes in cancer cell nuclei. To accurately assess cancer cell viability, we should have used stains effective for the evaluation of mitochondrial function, e.g., nicotinamide adenine dinucleotide staining, on the cancer cell clusters, but we identified at least two cancer cell clusters that seemed definitely viable in the hematoma, even on hematoxylin and eosin staining [9]. We believe these two isolated cancer cell clusters were present near the main breast cancer and judged viable; it is possible that they would have shown similar viability even if they had been present distant from the original cancer cell focus.

We cannot rule out the possibility that the hematoma surrounding the cancer cell clusters may be related to the needle tract recurrence. It, however, is very natural for researchers to conclude that the hematoma presence did not affect the viability of the two cancer cell clusters in this case because almost all reported needle tract recurrence cases have developed their recurrent foci not in the vicinity of the original tumors, but near or at the skin puncture site on core needle biopsy [10-12]. Furthermore, we have encountered a case of pancreatic cancer with subclinical needle tract seeding near the primary lesion without hematoma formation [13], supporting the idea that hematoma presence is not imperative for cancer cell migration with retained viability.

Naturally, tubule-forming and scirrhous-type invasive ductal carcinomas can also cause needle tract recurrence. We, however, speculate that migrated cancer cell volumes should be much lower in these breast cancer phenotypes than those with expansive-type growth patterns. In fact, we could identify a relatively large volume of migrated cancer cell clusters in this case. In this case, pathological findings easily bring us to the idea that a partial mastectomy was sufficient to remove the migrated cancer cell clusters due to their short distance from the primary tumor. Attending physicians, however, should have increased vigilance, based on this aspect of needle tract recurrence, in choosing the skin puncture site during core needle biopsy and whether to resect the needle tract.

## Conclusions

Diagnostic physicians should perform core needle biopsies for any kind of breast cancer subtype under the full consideration of needle tract recurrence. Many studies, however, reported that the vast majority of needle tract recurrence cases showed recurrent foci in solid expansive forms. Although this study is based on only one case, the pathological findings further support the importance of diagnostic physicians' awareness of expansive-type breast cancers to avoid needle tract recurrence on core needle biopsy.
